# In *Escherichia coli* Ammonia Inhibits Cytochrome *bo*_3_ But Activates Cytochrome *bd*-I

**DOI:** 10.3390/antiox10010013

**Published:** 2020-12-25

**Authors:** Elena Forte, Sergey A. Siletsky, Vitaliy B. Borisov

**Affiliations:** 1 Department of Biochemical Sciences, Sapienza University of Rome, P.le A. Moro 5, 00185 Rome, Italy; 2 Belozersky Institute of Physico-Chemical Biology, Lomonosov Moscow State University, Leninskie Gory, 119991 Moscow, Russia; siletsky@belozersky.msu.ru

**Keywords:** bacteria, redox enzymes, respiratory oxidases, ammonia, environmental stressor

## Abstract

Interaction of two redox enzymes of *Escherichia coli*, cytochrome *bo*_3_ and cytochrome *bd*-I, with ammonium sulfate/ammonia at pH 7.0 and 8.3 was studied using high-resolution respirometry and absorption spectroscopy. At pH 7.0, the oxygen reductase activity of none of the enzymes is affected by the ligand. At pH 8.3, cytochrome *bo*_3_ is inhibited by the ligand, with 40% maximum inhibition at 100 mM (NH_4_)_2_SO_4_. In contrast, the activity of cytochrome *bd*-I at pH 8.3 increases with increasing the ligand concentration, the largest increase (140%) is observed at 100 mM (NH_4_)_2_SO_4_. In both cases, the effector molecule is apparently not NH_4_^+^ but NH_3_. The ligand induces changes in absorption spectra of both oxidized cytochromes at pH 8.3. The magnitude of these changes increases as ammonia concentration is increased, yielding apparent dissociation constants *K*_d*app*_ of 24.3 ± 2.7 mM (NH_4_)_2_SO_4_ (4.9 ± 0.5 mM NH_3_) for the Soret region in cytochrome *bo*_3_, and 35.9 ± 7.1 and 24.6 ± 12.4 mM (NH_4_)_2_SO_4_ (7.2 ± 1.4 and 4.9 ± 2.5 mM NH_3_) for the Soret and visible regions, respectively, in cytochrome *bd*-I. Consistently, addition of (NH_4_)_2_SO_4_ to cells of the *E. coli* mutant containing cytochrome *bd*-I as the only terminal oxidase at pH 8.3 accelerates the O_2_ consumption rate, the highest one (140%) being at 27 mM (NH_4_)_2_SO_4_. We discuss possible molecular mechanisms and physiological significance of modulation of the enzymatic activities by ammonia present at high concentration in the intestines, a niche occupied by *E. coli*.

## 1. Introduction

Cytochrome *bo*_3_ and cytochrome *bd*-I are terminal oxidases in the aerobic respiratory chain of *Escherichia coli* [1]. Both enzymes catalyze the same redox reaction, the electron transfer from ubiquinol-8 to molecular oxygen giving rise to ubiquinone-8 and water [2,3]. In both cases this reaction is coupled to the formation of an electrochemical proton gradient across the bacterial cytoplasmic membrane [4,5,6]. Nevertheless, the *bo*_3_ oxidase shows a higher energy transduction efficiency than the *bd*-I oxidase because the former utilizes the proton pumping mechanism [4,7].

The 3D structures of the proteins were determined [8,9,10]. Each one is composed of four different subunits, however the cytochromes are structurally and evolutionarily unrelated. Cytochrome *bo*_3_ is a member of type A-1 of the heme-copper oxidase superfamily [11,12,13,14,15,16]. It carries the ubiquinol binding site, two hemes, *b* and *o*_3_, and a copper ion [17]. The latter, denoted Cu_B_, forms together with heme *o*_3_ a binuclear site in which the oxygen chemistry takes place. The *bd*-type oxidases form their own family, distinct from the heme-copper superfamily, and the *E. coli* cytochrome *bd*-I belongs to the L subfamily of that family [3,18]. The *bd*-I enzyme has no copper but contains a binding site for ubiquinol and three hemes, *b*_558_, *b*_595_, and *d*. Heme *d* serves as the site for the O_2_ reduction reaction.

Cytochrome *bo*_3_ and cytochrome *bd*-I are expressed in *E. coli* under normal and low aeration conditions, respectively [19]. This is consistent with the fact that *bo*_3_ is a low-oxygen-affinity oxidase [20], whereas *bd*-I is a high-oxygen-affinity oxidase [21]. The oxidases *bo*_3_ and *bd*-I differ in their sensitivity to small ligands. Cytochrome *bo*_3_ was shown to be much more sensitive to NO, H_2_S and cyanide than cytochrome *bd*-I [22,23,24,25,26,27]. The situation seems to be the opposite only in relation to the inhibition of the oxidases by CO [28]. The *bd*-I enzyme also contributes to the protection of *E. coli* against oxidative and nitrosative stress, playing an active antioxidant role in scavenging peroxynitrite and H_2_O_2_ [29,30,31,32]. The fact that the *bd*-type oxidase endows microbes with resistance to the toxic small molecules may explain why this respiratory enzyme is so abundant among bacterial pathogens. Since the *bd* protein is found only in prokaryotes, it may become a suitable target for next-generation antimicrobials [33].

*E. coli* is a consistent inhabitant of the intestinal tract of humans and warm-blooded animals. It is known that the intestines, particularly the large intestine lumen, reveal very high concentrations of ammonia, being in the millimolar range [34]. This raises the question of whether this ligand affects the functioning of the bacterial terminal oxidases. In this work, we have examined the effect of ammonia on oxygen consumption of cytochromes *bo*_3_ and *bd*-I of *E. coli* (at the level of both isolated enzymes and intact cells) and absorption spectra of the enzymes. To our knowledge, the effect of this ligand on a terminal quinol oxidase has never been studied.

## 2. Materials and Methods

### 2.1. Reagents and Purification of Cytochromes bd-I and bo_3_ from E. coli

Tris Base was purchased from Fisher BioReagents. Other chemicals were purchased from Sigma-Aldrich. Cytochromes *bd*-I and *bo*_3_ were isolated from the *E. coli* strains GO105/pTK1 and GO105/pJRhisA, respectively, as described by [35,36,37]. In the case of cytochrome *bd*-I, the fractions with an absorbance ratio of *A*_412_/*A*_280_ ≥ 0.7 eluted from a DEAE Sepharose Fast Flow anion exchange column were pooled and concentrated [36]. Cytochrome *bo*_3_ preparations were a kind gift of Marina Verkhovskaya (University of Helsinki). Being a His-tagged fusion protein, cytochrome *bo*_3_ was purified by immobilized metal chelate affinity chromatography on Ni-NTA Agarose. The fractions eluted from the column containing pure four-subunit cytochrome *bo*_3_ were pooled and concentrated [37]. The sample quality was evaluated by measuring enzyme activity and absorption spectra. All prepared samples showed high oxygen reductase activity (*V*_max_ of about 150 mol O_2_/mol cytochrome *bd*-I/s and 60 mol O_2_/mol cytochrome *bo*_3_/s in the presence of the electron donor/mediator couple 10 mM dithiothreitol (DTT) and 0.25 mM 2,3-dimethoxy-5-methyl-6-(3-methyl-2-butenyl)-1,4-benzoquinone (Q_1_) at pH 7.0) and characteristic absorption spectra both “as prepared” and dithionite-reduced. The concentration of cytochrome *bd*-I was determined from the dithionite reduced-*minus*-“as prepared” difference absorption spectra using Δ*ε*_628-607_ of 10.8 mM^−1^ cm^−1^ [38]. Cytochrome *bo*_3_ concentration was estimated from the Soret absorption band of the oxidized enzyme using ε_407_ of 182 mM^−1^ cm^−1^ [39].

### 2.2. Measurement Techniques and Assay Conditions.

O_2_ consumption of cytochromes *bd*-I and *bo*_3_ was measured using an Oxygraph-2k high-resolution respirometer (Oroboros Instrument, Innsbruck, Austria) equipped with two 1.5-mL chambers. UV-visible absorption spectra were recorded in an Agilent Cary 60 UV-Vis spectrophotometer. Assays were performed at 25 °C in 100 mM Tris-phosphate (pH 8.3) or 100 mM potassium phosphate (pH 7.0) buffer containing 0.1 mM EDTA, 2.5 μg/mL catalase, 10 mM DTT, 0.25 mM Q_1_, and either 0.05% *N*-lauroyl-sarcosine (cytochrome *bd*-I) or 0.02% dodecyl-β-d-maltoside (cytochrome *bo*_3_). The concentrations of cytochrome *bo*_3_ and cytochrome *bd*-I used in the oxygraphic measurements were 20 nM and 7.8 nM at pH 8.3, and 12 nM and 3.9 nM at pH 7.0 respectively. In the spectroscopic measurements, the concentrations of cytochrome *bo*_3_ and cytochrome *bd*-I were 4.8 μM and 3.2 μM respectively. The pH of the stock solutions of (NH_4_)_2_SO_4_ and K_2_SO_4_ was adjusted to the desired values (8.3 or 7.0). To generate the reduced state of cytochrome *bo*_3_ or cytochrome *bd-*I, a few grains of solid sodium dithionite were added. The oxidized state of cytochrome *bd-*I was produced by incubating the “as isolated” enzyme with 33 μM tetrachloro-1,4-benzoquinone for 10 min [40]. To remove excess oxidant, the sample was centrifuged at 4 °C and the yellow pellet discarded.

### 2.3. Data Analysis

Data analysis was carried out using Origin (OriginLab Corporation). To compare the (NH_4_)_2_SO_4_ titration data obtained in oxygraphic and spectroscopic experiments, they were fitted to the standard hyperbolic equation *y* = *A*_max_·*x*/(*K*_d*app*_ + *x*) using a built-in approximation function (“hyperbola function”) in “advanced fitting tool” in the Origin program. *A*_max_ and *K*_d*app*_ parameters were allowed to vary. *K*_d*app*_ is an *apparent* dissociation constant. In oxygraphic experiments, *A*_max_ is either a theoretical maximum percent inhibition (cytochrome *bo*_3_) or a theoretical maximum percent activity (cytochrome *bd*-I). In spectroscopic experiments, *A*_max_ is a theoretical maximum absorption change. *R*-square (*R*^2^) and standard deviation reported by the Origin program are shown in the figure legends. Since the high ionic strength may affect the activity of an enzyme [41], in order to take into account this possible effect, K_2_SO_4_ at the same concentration was added to the enzyme for each condition as a control.

## 3. Results

### 3.1. Effect of (NH_4_)_2_SO_4_ on O_2_ Reductase Activity of E. coli Cytochrome bo_3_

**Figure 1:**
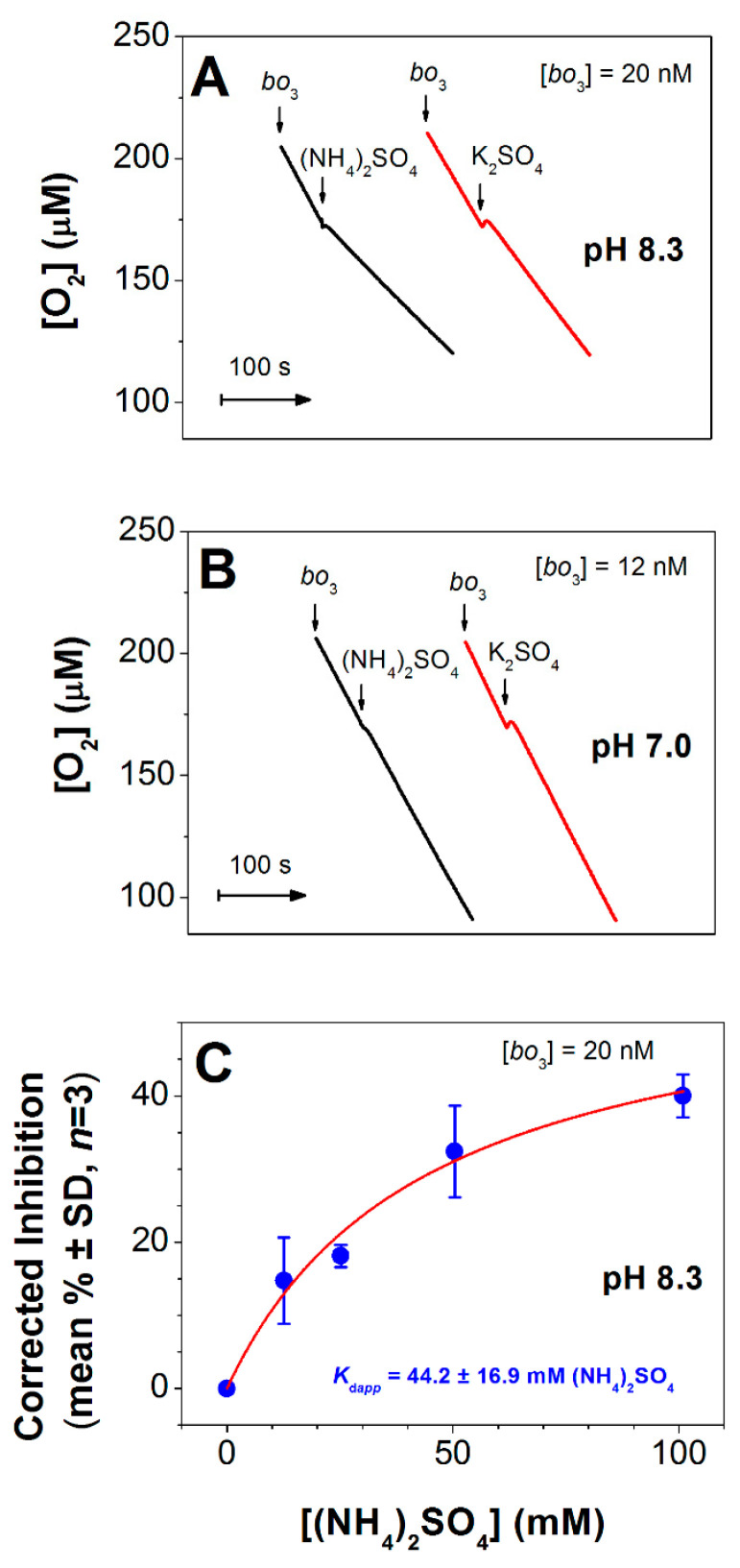
Effect of (NH_4_)_2_SO_4_ on cytochrome *bo*_3_ activity. (**A**) O_2_ consumption traces at pH 8.3. 50 mM (NH_4_)_2_SO_4_ inhibits the enzyme by 51% whereas 50 mM K_2_SO_4_ inhibits the enzyme by 18% (*n* = 3 for each experimental condition). (**B**) O_2_ consumption traces at pH 7.0. Neither 50 mM (NH_4_)_2_SO_4_ nor 50 mM K_2_SO_4_ affects the oxidase activity. (**C**) Percentage inhibition of O_2_ reductase activity of cytochrome *bo*_3_ measured at pH 8.3 at increasing concentration of (NH_4_)_2_SO_4_. The effect of increasing ionic strength on enzyme activity is taken into account for each data point by subtracting the percent inhibition value in the presence of K_2_SO_4_ (control) from that in the presence of (NH_4_)_2_SO_4_ at the same concentration. Experimental data (filled circles) are shown together with their best fit (solid line) to the hyperbolic equation (see Materials and Methods), giving a maximum percent inhibition value *A*_max_ of 58.4 ± 10.1%, and *K*_d*app*_ of 44.2 ± 16.9 mM (NH_4_)_2_SO_4_ (8.9 ± 3.4 mM NH_3_) (mean ± standard deviation, *n* = 3, *R*^2^ = 0.83663). O_2_ reductase activity of cytochrome *bo*_3_ is sustained by 10 mM DTT and 0.25 mM Q_1_. Enzyme, 20 nM (**A**,**C**) or 12 nM (**B**). In the absence of (NH_4_)_2_SO_4_, *V*_max_ values are 28 ± 2 and 60 ± 5 mol O_2_/mol enzyme/s at pH 8.3 and 7.0, respectively.

The effect of (NH_4_)_2_SO_4_ on the O_2_-reductase activity of the isolated cytochrome *bo*_3_ from *E. coli* was examined by measuring the O_2_ consumption rates before and after addition of the effector at pH 8.3 or 7.0. Figure 1A shows that at pH 8.3 the addition of 50 mM (NH_4_)_2_SO_4_ rapidly inhibits the O_2_ reductase activity of cytochrome *bo*_3_ by 51%. To take into account the effect of increasing ionic strength on enzyme activity, K_2_SO_4_ was added to the oxidase under the same conditions as a control. As shown in Figure 1A, 50 mM K_2_SO_4_ at pH 8.3 inhibits cytochrome *bo*_3_ to a much lesser extent (by 18%). At pH 7.0 (NH_4_)_2_SO_4_ does not inhibit the O_2_ reductase activity of the *bo*_3_ enzyme (Figure 1B). Figure 1C shows that with the increase in (NH_4_)_2_SO_4_ concentration, the inhibitory effect at pH 8.3 is progressively increased. The maximum inhibition observed (after subtraction of the corresponding control value with K_2_SO_4_) was 40% at 100 mM (NH_4_)_2_SO_4_.

We also studied the effect of (NH_4_)_2_SO_4_ on O_2_ consumption by *E. coli* mutant cells expressing cytochrome *bo*_3_ as a single terminal oxidase. Supplementary Figures S1B and S2 show that within the limits of the experimental error the addition of (NH_4_)_2_SO_4_ up to 27 mM at pH 8.3 caused no significant change in the respiration of the intact cells.

### 3.2. Effect of (NH_4_)_2_SO_4_ on Absorption Spectra of E. coli Cytochrome bo_3_

**Figure 2:**
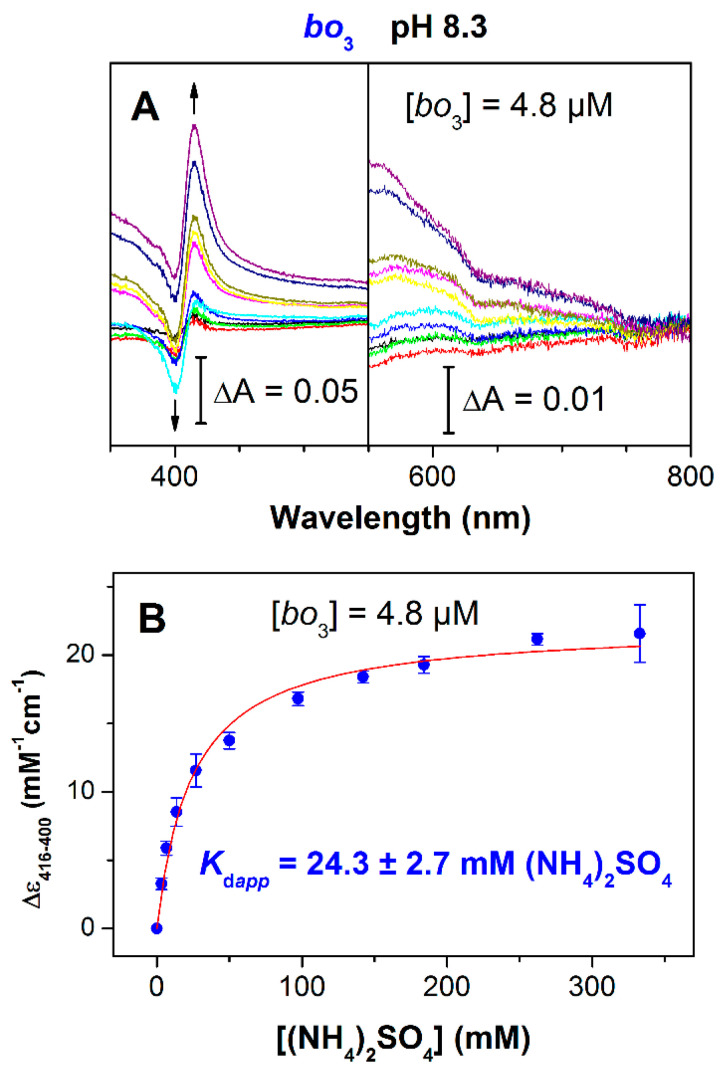
Absorbance changes of oxidized cytochrome *bo*_3_ induced by (NH_4_)_2_SO_4_. (**A**) Double difference absorption spectra of the isolated cytochrome *bo*_3_ (4.8 μM): each spectrum is a difference between two difference spectra, (NH_4_)_2_SO_4_-treated oxidized minus oxidized and K_2_SO_4_-treated oxidized minus oxidized at the same concentration of the sulfate. The arrows depict the direction of absorbance changes at increasing [(NH_4_)_2_SO_4_]. (**B**) Absorbance changes measured at 416–400 nm as a function of [(NH_4_)_2_SO_4_]. Experimental data (*filled circles*) are shown together with their best fit (*solid line*) to the hyperbolic equation (see Materials and Methods), giving the value for maximum absorption change at 416–400 nm *A*_max_ of 22.1 ± 0.6 mM^−1^cm^−1^, and *K*_d*app*_ of 24.3 ± 2.7 mM (NH_4_)_2_SO_4_ (4.9 ± 0.5 mM NH_3_) (mean ± standard deviation, *n* = 3, *R*^2^ = 0.98947).

The finding that (NH_4_)_2_SO_4_ can inhibit cytochrome *bo*_3_ pushed us to explore the effect of ammonia on absorption spectra of the isolated cytochrome *bo*_3_. Figure 2A shows the spectral changes induced by the addition of (NH_4_)_2_SO_4_ at millimolar concentrations to the oxidized cytochrome *bo*_3_ at pH 8.3. The ammonia-induced spectrum showed a red shift of the enzyme Soret band with a maximum at 416 nm and a minimum at 400 nm. In the visible spectrum, some weak intensity broad bands were displayed, the most pronounced of which was a band with a minimum around 630 nm. The spectral changes caused by the ligand are possibly due to its binding to the heme *o*_3_-Cu_B_ binuclear site. The observed changes could be also due to a small reduction of the enzyme. However, the reduced-minus-oxidized spectrum in the Soret region of cytochrome *bo*_3_ showed a maximum at 428–430 nm [39,42] rather than 416 nm. The magnitude of the Soret spectral changes increased as (NH_4_)_2_SO_4_ concentration was increased (Figure 2B). Analysis of the titration curve yields *K*_d*app*_ of 24.3 ± 2.7 mM (NH_4_)_2_SO_4_ and the value for maximum absorption change at 416–400 nm *A*_max_ of 22.1 ± 0.6 mM^−1^cm^−1^. It has to be noted that the titration profile (Figure 2B) is similar to that for the plot of percent inhibition versus (NH_4_)_2_SO_4_ (Figure 1C). The addition of (NH_4_)_2_SO_4_ to the dithionite-reduced cytochrome *bo*_3_ under identical conditions brought about no spectral change.

### 3.3. Effect of (NH_4_)_2_SO_4_ on O_2_ Reductase Activity of E. coli Cytochrome bd-I

Next, we studied the influence (NH_4_)_2_SO_4_ on the O_2_-reductase activity of the isolated cytochrome *bd*-I from *E. coli* under the same conditions as used for the *bo*_3_ oxidase. We found that in contrast to cytochrome *bo*_3_, cytochrome *bd*-I is not inhibited by millimolar concentrations of (NH_4_)_2_SO_4_ at pH 8.3. Furthermore, under these conditions, activation of the catalytic activity of the enzyme was observed.

**Figure 3:**
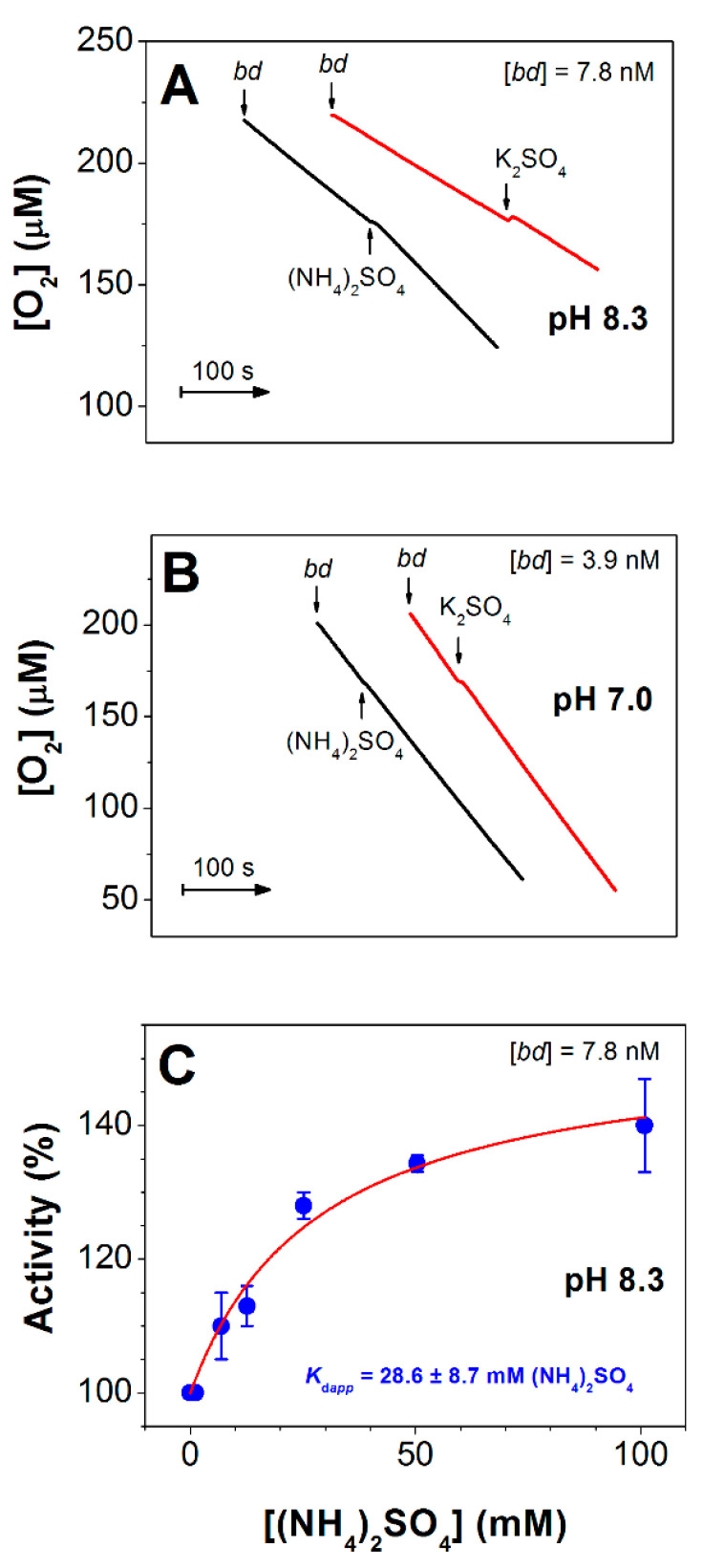
Effect of (NH_4_)_2_SO_4_ on cytochrome *bd-*I activity. (**A**) O_2_ consumption traces at pH 8.3. A total of 25 mM (NH_4_)_2_SO_4_ increases O_2_ consumption rate of the enzyme by 39% whereas 25 mM K_2_SO_4_ increases the rate by 11% (*n* = 3 for each experimental condition). (**B**) O_2_ consumption traces at pH 7.0. Neither 25 mM (NH_4_)_2_SO_4_ nor 25 mM K_2_SO_4_ affects the oxidase activity. (**C**) Dependence of O_2_ reductase activity of cytochrome *bd-*I measured at pH 8.3 on the concentration of (NH_4_)_2_SO_4_. Values are expressed with reference to the activity measured before sulfate addition taken as 100%. The effect of increasing ionic strength on enzyme activity is taken into account for each data point by subtracting the activity value in the presence of K_2_SO_4_ (control) from that in the presence of (NH_4_)_2_SO_4_ at the same concentration. Experimental data (filled circles) are shown together with their best fit (solid line) to the hyperbolic equation (see Materials and Methods), giving a maximum activity value *A*_max_ of 152.9 ± 6.3%, and *K*_d*app*_ of 28.6 ± 8.7 mM (NH_4_)_2_SO_4_ (5.7 ± 1.7 mM NH_3_) (mean ± standard deviation, *n* = 3, *R*^2^ = 0.92789). O_2_ reductase activity of cytochrome *bd-*I is sustained by 10 mM DTT and 0.25 mM Q_1_. Enzyme, 7.8 nM (**A**,**C**) or 3.9 nM (**B**). In the absence of (NH_4_)_2_SO_4_, *V*_max_ values are 30 ± 6 and 152 ± 15 mol O_2_/mol enzyme/s at pH 8.3 and 7.0, respectively.

As shown in Figure 3A, at pH 8.3 the addition of 25 mM (NH_4_)_2_SO_4_ increased the rate of O_2_ consumption of cytochrome *bd*-I by 39%. In the control with 25 mM K_2_SO_4_, the increase in the rate was significantly lower (by 11%). At pH 7.0, there was no effect of (NH_4_)_2_SO_4_ on the O_2_ reductase activity of cytochrome *bd*-I (Figure 3B). At pH 8.3, the O_2_ reductase activity of cytochrome *bd*-I increased with an increase in ammonia concentration (Figure 3C). Maximum activation in enzyme activity, 140%, was observed following the addition of 100 mM (NH_4_)_2_SO_4_ (after subtraction of the control with K_2_SO_4_).

We also studied the effect of (NH_4_)_2_SO_4_ on O_2_ consumption by *E. coli* mutant cells containing cytochrome *bd*-I as the only terminal oxidase. Supplementary Figures S1A and S2 show that at pH 8.3 the addition of (NH_4_)_2_SO_4_ up to 27 mM increased respiration of the intact cells. Maximum acceleration of the O_2_ consumption rate (140%) was observed at 27 mM (NH_4_)_2_SO_4_. In the control with K_2_SO_4_ added at the same concentrations, there was no significant change in O_2_ consumption by the cells.

### 3.4. Effect of (NH_4_)_2_SO_4_ on Absorption Spectra of E. coli Cytochrome bd-I

**Figure 4:**
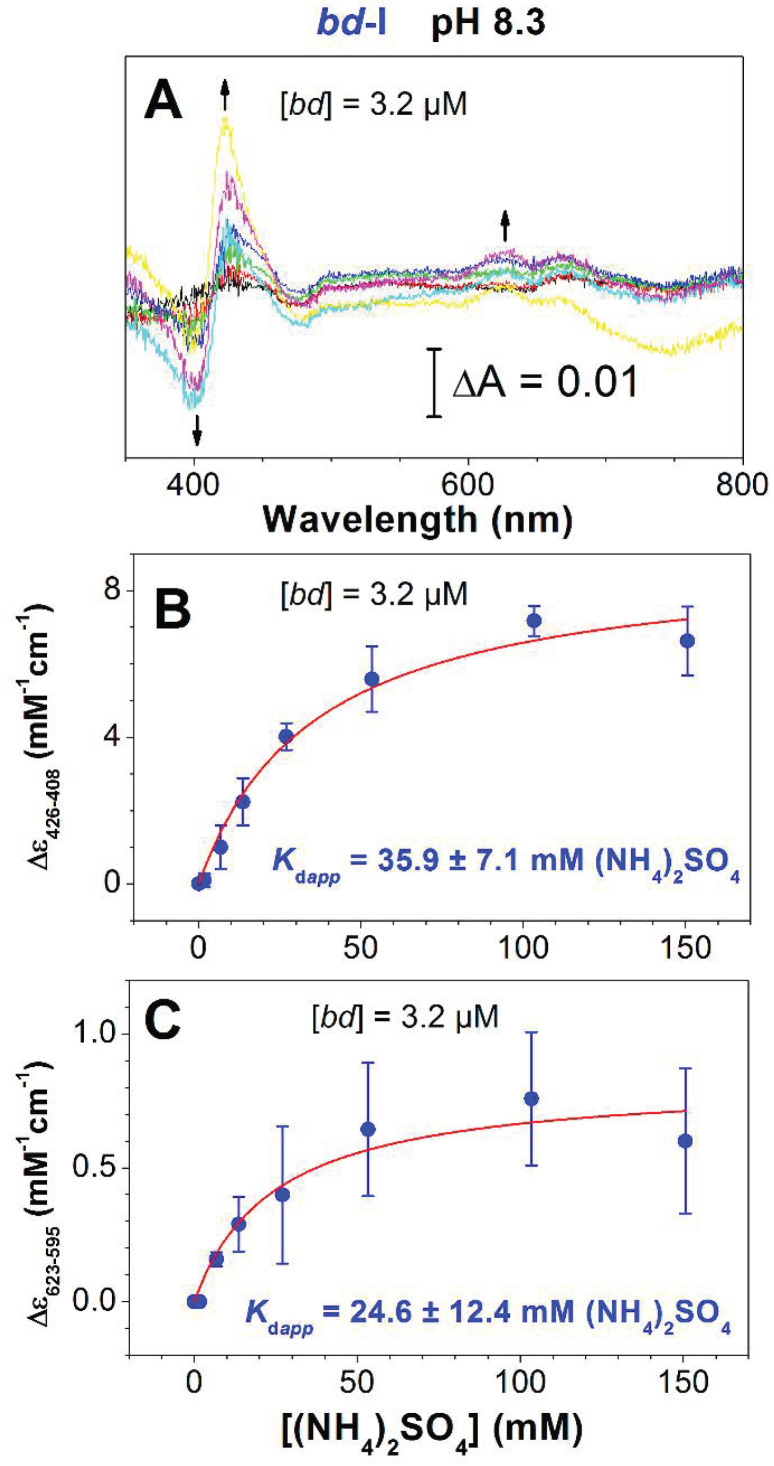
Absorbance changes of oxidized cytochrome *bd-*I induced by (NH_4_)_2_SO_4_. (**A**) Double difference absorption spectra of the isolated cytochrome *bd-*I (3.2 μM): each spectrum is a difference between two difference spectra, (NH_4_)_2_SO_4_-treated oxidized minus oxidized and K_2_SO_4_-treated oxidized minus oxidized at the same concentration of the sulfate. The arrows depict the direction of absorbance changes at increasing [(NH_4_)_2_SO_4_]. (**B**,**C**) Absorbance changes measured at 426–408 and 623–595 nm as a function of (NH_4_)_2_SO_4_. Experimental data (filled circles) are shown together with their best fits (solid lines) to the hyperbolic equation (see Materials and Methods), giving the values for maximum absorption changes at 426–408 and 623–595 nm *A*_max_ of 8.9 ± 0.6 and 0.8 ± 0.1 mM^−1^cm^−1^ and *K*_d*app*_ of 35.9 ± 7.1 and 24.6 ± 12.4 mM (NH_4_)_2_SO_4_ (7.2 ± 1.4 and 4.9 ± 2.5 mM NH_3_), respectively (mean ± standard deviation, *n* = 3; *R*^2^ = 0.94547 (Soret region), *R*^2^ = 0.69231 (visible region)).

Finally, we found that ammonia affects the absorption spectrum of the isolated cytochrome *bd*-I in the fully oxidized state. Figure 4A displays the spectral changes caused by the addition of (NH_4_)_2_SO_4_ at millimolar concentrations to the oxidized *bd*-I enzyme at pH 8.3.

In the Soret region, the ammonia-induced spectrum showed a red shift with a maximum at about 423–428 nm and a minimum around 395–408 nm. In the near-IR region, there were two maxima at ~623 and ~673 nm, and a broad minimum at ~740 nm. The changes may report the interaction of ammonia with the ferric heme *d*. The magnitude of the changes increased with increasing concentrations of (NH_4_)_2_SO_4_. Analysis of the absorption titration curves measured at 426–408 and 623–595 nm (Figure 4B,C) yields *K*_d*app*_ of 35.9 ± 7.1 and 24.6 ± 12.4 mM (NH_4_)_2_SO_4_, respectively. The titration profile (Figure 4B,C) is similar to that for the plot of percent enzyme activity versus (NH_4_)_2_SO_4_ (Figure 3C).

No spectral change was observed, when under the same conditions, (NH_4_)_2_SO_4_ was added to cytochrome *bd*-I in the dithionite-reduced state.

## 4. Discussion

### 4.1. Proposed Mechanism for Inhibition of Cytochrome bo_3_ by Ammonia

The interaction of ammonia with a quinol oxidase has not been investigated before. Recently, von der Hocht et al. observed that in the presence of 20 mM (NH_4_)_2_SO_4_ at pH 9 the activity of the isolated *aa*_3_-type cytochrome *c* oxidase from *Paracoccus denitrificans* sustained by ascorbate, *N*,*N*,*N’*,*N’*-tetramethyl-*p*-phenylenediamine and cytochrome *c* decreased by 22% [43]. They also reported that at pH 9 the addition of ammonia to the H_2_O_2_-generated **F** state led to its conversion into a novel **P** state called **P_N_** [43]. **P** and **F** are two transient ferryl intermediates formed sequentially during the catalytic cycle of both heme-copper and *bd*-type terminal oxidases [44,45,46,47,48,49,50]. In the case of cytochrome *c* oxidase, spectrally similar artificial **P** and **F** intermediates can also be produced by the addition of H_2_O_2_ at different concentrations to the enzyme in the oxidized (**O**) state at alkaline pH [51,52]. In the new **P_N_** state [43], ammonia binds to Cu_B_, as shown by resonance Raman spectroscopy [53].

Here, we showed that the O_2_ consumption of the isolated *E. coli* cytochrome *bo*_3_, sustained by DTT and Q_1_, was inhibited by (NH_4_)_2_SO_4_ at pH 8.3 (Figure 1A). At the maximum concentration of (NH_4_)_2_SO_4_ used (100 mM), the enzyme activity decreased by 40% (Figure 1C). The inhibition was not observed at pH 7.0. The p*K*_a_ of ammonium in aqueous solution is known to be 9.25 at 25 °C. Using the Henderson-Hasselbalch equation one can calculate that when 100 mM of (NH_4_)_2_SO_4_ is added to the sample at pH 8.3 [NH_4_^+^] = 179.85 mM and [NH_3_] = 20.15 mM, whereas at pH 7.0 [NH_4_^+^] = 198.88 mM and [NH_3_] = 1.12 mM. In other words, by shifting the pH from 7.0 to 8.3, [NH_3_] increases 18 times, while [NH_4_^+^] does not change significantly (decreases only 1.1 times). Thus, we can conclude that it is ammonia rather than the ammonium ion that inhibits cytochrome *bo*_3_.

At pH 8.3, ammonia caused a red shift in the Soret band of the oxidized cytochrome *bo*_3_ (Figure 2A). The magnitude of the absorption changes increased with increasing the ligand concentration giving *K*_d*app*_ of 24.3 ± 2.7 mM (NH_4_)_2_SO_4_ that corresponds to 4.9 ± 0.5 mM NH_3_ (Figure 2B). The titration profile was similar to that for the inhibition of the enzyme activity by ammonia (Figure 1C). Ingledew et al. earlier reported that the addition of cyanide induces a red shift in the Soret band of the oxidized cytochrome *bo*_3_ [54]. The binding of a ligand to the high-spin heme brings about a red shift of the Soret band in enzyme absorption spectra [1,54,55]. On the contrary, no absorption change or a small blue shift in the Soret band was observed when a ligand binds to Cu_B_ [1,56]. Thus, we suggest that ammonia binds to heme *o*_3_. This conclusion is supported by the fact that the ligand-induced red shift in the Soret band was accompanied by the loss of the broad charge transfer band around 630 nm (Figure 2A). The 630-nm band is characteristic of the fully oxidized binuclear site in which heme *o*_3_ is in a high-spin state [54]. The decay of this band suggests the conversion of the high-spin heme *o*_3_ into the low-spin ammonia complex. As a ferric heme usually binds an anion, we propose that NH_3_ binds to heme *o*_3_ in the form of NH_2_^−^ with the release of H^+^. Both cyanide and azide can bridge between the ferric heme *o*_3_ and cupric Cu_B_ forming the following structures: Fe*_o_*_3_^3+^–C=N–Cu_B_^2+^ and Fe*_o_*_3_^3+^–N=N=N–Cu_B_^2+^, where Fe*_o_*_3_ is the heme *o*_3_ iron [55,57,58]. Compared to these two ligands, ammonia is a much smaller molecule. Rather, NH_3_ can be compared to a water/hydroxide molecule that is a natural ligand of Cu_B_ in several states of the catalytic cycle of heme–copper oxidases [59]. For this reason, NH_3_ cannot be a bridging ligand at the binuclear site since a distance is around 4–5 Å. However, the binding of two NH_3_ molecules at the oxidized binuclear site at a time, one to heme *o*_3_ and the other to Cu_B_, may occur. Indeed, NH_3_ is approximately the size of a water/hydroxide molecule. In some catalytic intermediates of a heme–copper oxidase, two molecules of H_2_O (or OH^−^) can bind simultaneously at the binuclear site [59,60,61]. It could also be true for ammonia. We propose to designate the ammonia adduct of the oxidized cytochrome *bo*_3_ as **N**.

Importantly, the binding of hydroxide (as opposed to water) with the oxidized heme at the binuclear site leads to the transition of the heme from the high-spin to the low-spin state [62]. This process is enhanced at alkaline pH. For instance, at pH 9, about 50% of the ferric heme *a*_3_ is hydroxide-ligated whereas at pH 6.5, no hydroxide is bound to the heme [63]. The spectral shift caused by ammonia (Figure 2A) is similar to the effect of the formation of low-spin complexes of the initial high-spin heme with anionic ligands at the binuclear site. We suggest that when ammonia binds to the oxidized heme at alkaline pH, the complex with deprotonated ammonia (NH_2_^−^) is formed, just as it happens with hydroxide. It has to be noted that a similar complex can be produced in cytochrome *c* nitrite reductase, before the release of the neutral ammonia, the final product of nitrite reduction, from the catalytic site [64]. Notably, the presence of a tyrosine residue near the heme is critically important. In cytochrome *c* nitrite reductase, the residue facilitates the transition of the heme from the high-spin to the low-spin state (via the stabilization of the radical form of the bound NH_2_^−^) and serves as a proton donor/acceptor [64]. Surprisingly, all heme–copper oxidases also contain a conserved tyrosine residue that is part of the binuclear site. The tyrosine is bound to Cu_B_ through a histidine ligand and is critical for the proton pumping function. At the same time, the structure of cytochrome *bd* that lacks the proton pump shows no tyrosine residue nearby heme *d* (or heme *b*_595_) [9,10,65]. This could explain the higher sensitivity of the *bo*_3_ oxidase to the inhibitory effect of ammonia as compared to the *bd* oxidase.

**Figure 5:**
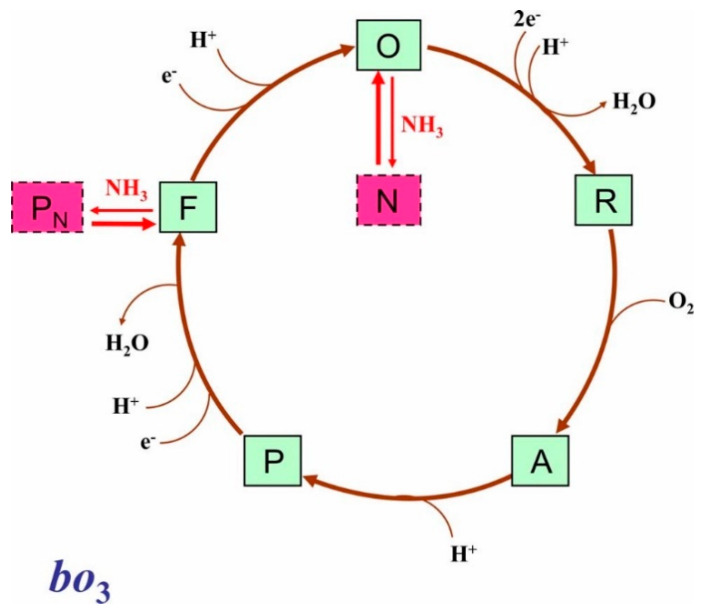
The possible effect of ammonia on the catalytic cycle of cytochrome *bo*_3_. Proposed catalytic intermediates **O** (*o*_3_^3+^–OH Cu_B_^2+^), **R** (*o*_3_^2+^ Cu_B_^+^), **A** (*o*_3_^2+^–O_2_ Cu_B_^+^), **P** (*o*_3_^4+^=O^2−^ Cu_B_^2+^–OH), and **F** (*o*_3_^4+^=O^2−^ Cu_B_^2+^) are shown. Possible redox and ligation state of the binuclear site in each intermediate is indicated in brackets. Only chemical protons are shown. Pumped protons are not shown for clarity. The two ferryl species **P** and **F** likely differ in the presence of an aromatic amino acid radical in **P**, as in the intermediate **P_M_** of cytochrome *c* oxidase [66]. NH_3_ presumably converts **F** into the **P_N_** state (*o*_3_^4+^=O^2−^ Cu_B_^2+^–NH_3_) and **O** into the ammonia complex **N** (*o*_3_^3+^–NH_2_^−^ Cu_B_^2+^ and/or *o*_3_^3+^– NH_2_^−^ Cu_B_^2+^–NH_3_), thereby leading to the inhibition of the oxidase activity.

Figure 5 shows a proposed molecular mechanism of inhibition of the catalytic activity of cytochrome *bo*_3_ by ammonia. The ligand binds to the catalytic intermediates **O** and **F** thereby blocking the oxygen reduction reaction cycle of the enzyme.

### 4.2. Proposed Mechanism for Ammonia-Induced Acceleration of the Cytochrome bd-I Activity

In contrast to cytochrome *bo*_3_, cytochrome *bd*-I at pH 8.3 was not inhibited by (NH_4_)_2_SO_4_ (Figure 3A). Furthermore, the addition of the ligand led to an increase in enzyme activity. The highest enhancement of the rate of cytochrome *bd*-I-catalyzed reaction (140%) was achieved upon the addition of 100 mM (NH_4_)_2_SO_4_ (Figure 3C). The fact that at pH 7.0 (NH_4_)_2_SO_4_ did not affect the enzyme activity (Figure 3B) suggests that the activator was ammonia rather than the ammonium ion. Consistently, the addition of (NH_4_)_2_SO_4_ to intact cells of the *E. coli* mutant possessing cytochrome *bd*-I as the sole terminal oxidase at pH 8.3 increased cell respiration (Supplementary Figures S1A and S2). Maximum acceleration of the O_2_ consumption rate (140%) was observed at 27 mM (NH_4_)_2_SO_4_.

At pH 8.3, the addition of ammonia brought about spectral changes in the fully oxidized cytochrome *bd*-I, the amplitude of which increased with increasing the ligand concentration (Figure 4). The titration curves (Figure 4B,C) were similar to that of the ammonia-induced activity change (Figure 3C). Surprisingly, the ammonia-induced difference absorption spectra (Figure 4A) were similar to the difference absorption spectra recorded following addition of H_2_O_2_ to the fully oxidized cytochrome *bd*-I [67,68]. In the reaction product, heme *d* was in the ferryl state [69]. As in the case of cytochrome *c* oxidase [51,52], after the addition of excess peroxide to cytochrome *bd*-I, the two ferryl species, P and F, were probably produced. P discovered by [47] is a heme *d* ferryl porphyrin π-cation radical intermediate [49]. Thus, the H_2_O_2_-induced difference spectra reported in [67,68] likely reflect a mixture of P and F.

**Figure 6:**
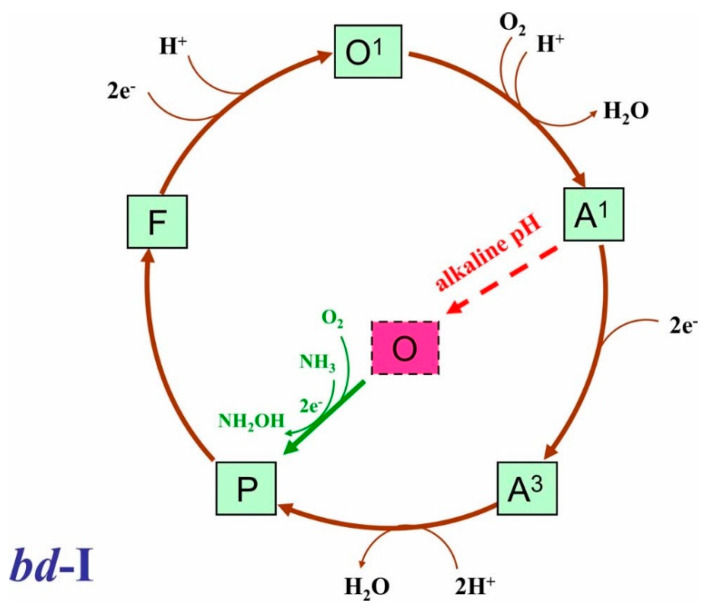
The possible effect of ammonia on the catalytic cycle of cytochrome *bd-*I. Enzyme catalytic intermediates **O^1^** (*b*_558_^2+^ *b*_595_^3+^ *d*^3+^–OH), **A^1^** (*b*_558_^3+^ *b*_595_^3+^ *d*^2+^–O_2_), **A^3^** (*b*_558_^2+^ *b*_595_^2+^ *d*^2+^–O_2_), **P** (*b*_558_^2+^ *b*_595_^3+^ d*^4+^=O^2^ where d*^4+^=O^2^ is a ferryl porphyrin π-cation radical [47,49]), and **F** (*b*_558_^3+^ *b*_595_^3+^ *d*^4+^=O^2-^) are shown. At alkaline pH, **A^1^** is probably converted into the fully oxidized form **O**. **O** is not involved in the catalytic cycle [72]. NH_3_ possibly promotes the formation of **P** from **O**, thereby leading to the acceleration of the oxidase activity. It is also possible that NH_3_ reacts with **O^1^** producing **F**. In the latter two reactions, NH_3_ serves as a two-electron donor being oxidized to NH_2_OH. The reaction of NH_3_ with **O^1^** is not shown for the sake of simplicity.

It is known that in the air-oxidized cytochromes *bd* from *E. coli* and *Azotobacter vinelandii* heme *d* is mostly in the oxygenated form [7,40,70,71]. This state is often called compound A^1^ (see Figure 6). Jünemann and Wrigglesworth reported that exposure of the *A. vinelandii* cytochrome *bd* in an air-oxidized state to alkaline pH leads to deoxygenation of heme *d* [71]. At alkaline pH, the heme *d* oxy-complex in the A^1^ state is destabilized and may decay to the oxidized (O) state (Figure 6).

A^1^ is a catalytic intermediate of cytochrome *bd*-I [73], whereas O does not participate in the catalytic cycle [72]. The conversion of A^1^ into O under alkaline conditions seems to correlate with the observation that the O_2_-reductase activity of cytochrome *bd*-I at pH 8.3 is lower than that at pH 7.0 (*V*_max_ of 30 ± 6 mol O_2_/mol enzyme/s at pH 8.3 versus 152 ± 15 mol O_2_/mol enzyme/s at pH 7.0). We hypothesize that the addition of ammonia to the oxidized cytochrome *bd*-I at pH 8.3 promotes the formation of P from O (Figure 6), thereby increasing the enzyme activity. NH_3_ may be oxidized to NH_2_OH serving as a two-electron donor in this reaction. NH_3_ could also react with the one electron-reduced enzyme (O^1^) with the production of F and NH_2_OH.

### 4.3. Possible Physiological Significance of the Difference between Cytochrome bo_3_ and Cytochrome bd-I in Sensitivity toward Ammonia for E. coli

Along with nitric oxide, carbon monoxide, and hydrogen sulfide, ammonia is considered as a “gasotransmitter” or endogenously generated gaseous signaling molecule [74]. A signaling role of NH_3_ in cultured rat astrocytes has been reported [75]. The molecule can move across the plasma membranes both passively [76] and actively via the Amt/Rh family of ammonium/ammonia transporting membrane proteins, involving the electrogenic transport mechanism [77,78]. Ammonia is a degradation product of proteins, peptides, amino acids, and urea. It is mainly produced by the gut microbiota and the gut, liver, kidney, and muscle cells. An adult human gut generates 4–10 g of NH_3_ daily [79]. *E. coli* is one of the most active ammonia-producers in the gut microbiota. Ammonia can also be recycled into amino acid synthesis [80]. NH_3_ is a potent infochemical in bacteria–bacteria interactions. It is able to induce oxidative stress responses and increase resistance to antibiotics thus playing a role in defense mechanisms against antimicrobials [81]. At high enough concentrations, ammonia is toxic to cells, especially to neurons. For this reason, the plasma concentration of ammonia in healthy adults is maintained in the range of 10–35 μM [82]. In the intestines, the total ammonia concentration depends on intestinal segment and diet but in general it is about 1000-times higher than in blood [34]. The highest ammonia concentration in the body (27.2 ± 17.5 mM) is reported to be in the large intestinal lumen [34]. Intestinal pH shows high variability depending on the intestinal segment, diet, and regional distribution of microbiota, and there are conditions in which the pH values are in the alkaline region [83,84]. For example, in the distal ileum, the median pH is 8.1 [85]. In light of the above, we suggest that the difference between the two quinol oxidases in the sensitivity toward ammonia can have a physiological significance for *E. coli* and other enterobacteria. In contrast to the heme–copper oxidase *bo*_3_ that is inhibited by NH_3_ at alkaline pH, the *bd*-I oxidase under the same conditions is not inhibited but activated by the ligand. Thus cytochrome *bd* can sustain bacterial respiration in the presence of high concentrations of not only sulfide [26,27,86], but also ammonia.

## 5. Conclusions

In summary, we investigated the sensitivity of two physiologically important respiratory cytochromes of *E. coli*, *bo*_3_ and *bd*-I, to ammonia at the level of both isolated enzymes and intact cells. It turned out that at pH 8.3 the isolated heme-copper *bo*_3_ oxidase is partly inhibited by NH_3_. Surprisingly, under the same conditions, the isolated copper-lacking *bd*-I enzyme is not only resistant to but also activated by this gaseous signaling molecule. Consistently, respiration of intact cells of the *E. coli* mutant that relies on cytochrome *bd*-I as the only terminal oxidase is accelerated by NH_3_. With such a unique trait, the *bd*-type redox enzyme may provide *E. coli* and perhaps other bacteria with the ability to maintain the aerobic energy metabolism in the gut and other ammonia-rich environments.

## Supplementary Materials

The following are available online at https://www.mdpi.com/2076-3921/10/1/13/s1, Figure S1: O_2_ consumption traces showing the effect of (NH_4_)_2_SO_4_ on the respiration of *E. coli* in respiratory mutants at pH 8.3, Figure S2: Effect of (NH_4_)_2_SO_4_ on the respiration of *E. coli* in respiratory mutants.

## Abbreviations

*K*_d*app*_	Apparent dissociation constant
DTT	Dithiothreitol
Q_1_	2,3-dimethoxy-5-methyl-6-(3-methyl-2-butenyl)-1,4-benzoquinone
