# Supplementary material for: In Escherichia coli Ammonia Inhibits Cytochrome bo3 But Activates Cytochrome bd-I
Source: Antioxidants (Basel). 2020 Dec 25;10(1):13. doi: 10.3390/antiox10010013 (PMC7824442; doi:10.3390/antiox10010013)
Supplement: Supplementary file 1 [file antioxidants-10-00013-s001.pdf]

Supplementary Materials for

Article

In *Escherichia coli* Ammonia Inhibits Cytochrome *bo*3 but Activates Cytochrome *bd*-I

Elena Forte, Sergey A. Siletsky and Vitaliy B. Borisov

Supplementary Figures

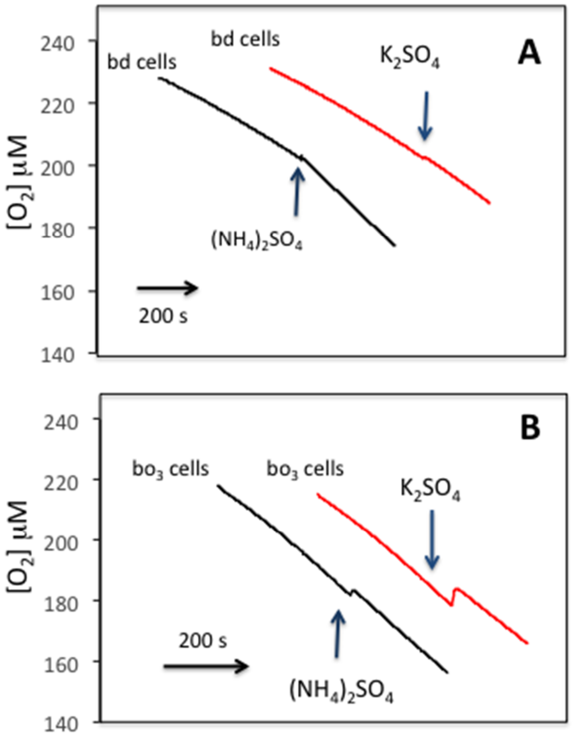

Supplementary Figure S1. O_2 consumption traces showing the effect of $(NH_4)_2SO_4$ on the respiration of *E. coli* in respiratory mutants at pH 8.3. **A)** The addition of 13 mM $(NH_4)_2SO_4$, but not 13 mM K_2SO_4, increases O_2 consumption by cell suspensions of the mutant strain expressing cytochrome *bd*-I as the only terminal oxidase (400 µl cells with OD_{600} = 2.3). **B**) Neither 13 mM $(NH_4)_2SO_4$ nor 13 mM K_2SO_4 affects respiration in the mutant strain expressing cytochrome *bo*3 as the sole oxidase (300 µl cells with OD_{600} = 2.15).

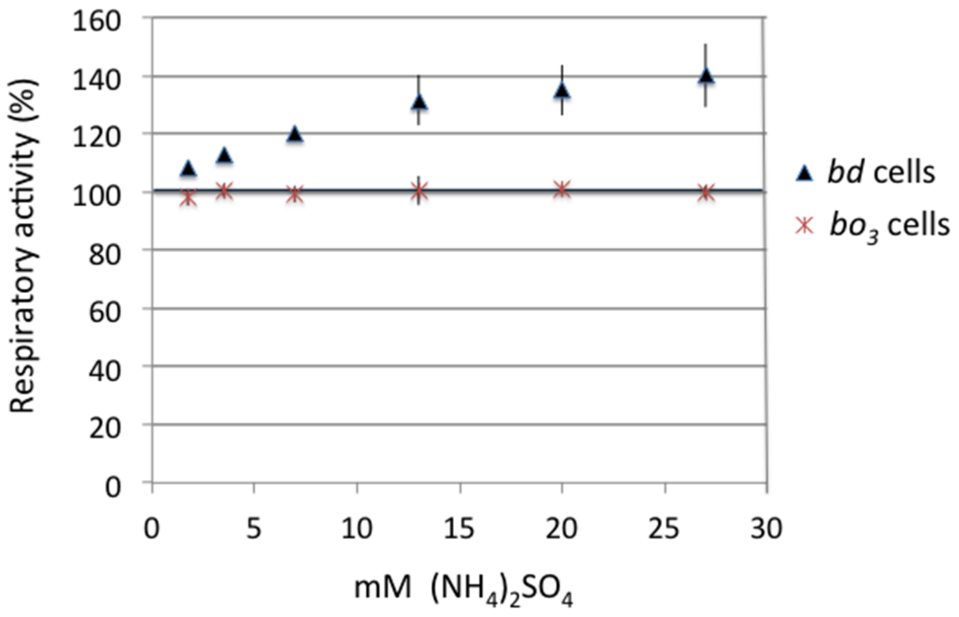

Supplementary Figure S2. Effect of $(NH_4)_2SO_4$ on the respiration of *E. coli* in respiratory mutants. The respiratory activity measured increased after the addition to mutant *E. coli* cells of $(NH_4)_2SO_4$ at increasing the ligand concentrations ranging from 1.75 mM to 27 mM. Data (mean ± standard deviation, n = 3) refer to the control activity measured before the addition of $(NH_4)_2SO_4$ (taken as 100%). Addition of K_2SO_4 in the same range of concentrations does not affect the respiratory activity of both mutants.

Supplementary Methods

Bacterial strains and growth conditions

The *E. coli* respiratory mutant strains used, derived from the K-12 derivative MG1655 (RKP5416), were TBE025 (MG1655 ΔcydB nuoB appB::kan) and TBE037 (MG1655 ΔappB nuoB cyoB::kan), respectively expressing cytochrome bo_3, and *bd*-I as the only terminal oxidase (mutants kindly given by Alex Ter Beek and Joost Teixeira de Mattos, University of Amsterdam).

E. coli cells were grown in 50 mL-Falcon tubes in 5 mL Luria Bertani (LB) medium supplemented with 30 µg/ mL kanamycin at 37 °C and 200 rpm.

Assay conditions

The respirometric assays were performed at 25 °C in 100 mM Tris-phosphate (pH 8.3).
